# Thromboinflammatory Biomarkers in Lymphomas: Linking Inflammation to Thrombosis Risk

**DOI:** 10.3390/ijms26052058

**Published:** 2025-02-26

**Authors:** Emilija Živković, Olivera Mitrović-Ajtić, Tijana Subotički, Jelena Ivanović, Vladimir Otašević, Dragoslava Đikić, Miloš Diklić, Milica Vukotić, Teodora Dragojević, Dejana Stanisavljević, Darko Antić, Vladan P. Čokić

**Affiliations:** 1 Institute for Medical Research, National Institute of the Republic of Serbia, University of Belgrade, 11000 Belgrade, Serbia; emilija.zivkovic@imi.bg.ac.rs (E.Ž.); oliveram@imi.bg.ac.rs (O.M.-A.); tijana@imi.bg.ac.rs (T.S.); dragoslava@imi.bg.ac.rs (D.Đ.); milos.diklic@imi.bg.ac.rs (M.D.); milica.tosic@imi.bg.ac.rs (M.V.); teodora.dragojevic@imi.bg.ac.rs (T.D.); 2 Lymphoma Center, Clinic for Hematology, University Clinical Center of Serbia, 11000 Belgrade, Serbia; jivanovic09@gmail.com (J.I.); darko.antic1510976@gmail.com (D.A.); 3 Parexel International, Durham, NC 27713, USA; vladimirota@hotmail.com; 4 Institute for Medical Statistics and Informatics, 11000 Belgrade, Serbia; dejana.stanisavljevic@med.bg.ac.rs; 5 Faculty of Medicine, University of Belgrade, 11000 Belgrade, Serbia

**Keywords:** non-Hodgkin lymphoma, Hodgkin lymphoma, thrombosis, inflammation, cytokines

## Abstract

Thrombosis is a critical complication in lymphomas, driven by chronic inflammation. To observe this systemic mechanism, we evaluated inflammatory cytokines, neutrophil and monocyte activation, and platelet function in diffuse large B-cell lymphoma (DLBCL), follicular lymphoma (FL), and Hodgkin lymphoma (HL), with and without thrombosis using ELISA and flow cytometry according to laboratory and clinical data. Interleukin-1β was elevated across lymphomas and inversely correlated with the Khorana score for venous thromboembolism, while increased tumor necrosis factor-alpha (TNF-α) was inversely associated with the International Prognostic Index (IPI) in thrombosis-associated lymphomas. Neutrophil activation was increased in DLBCL, while elevated neutrophil extracellular traps (NETs) biomarkers were inversely consistent with thrombosis and the ThroLy score. NETs were elevated in HL. Classical monocytes were increased in all lymphoma subtypes, with intermediate and tissue factor (TF)-carrying monocytes elevated in DLBCL and HL. Platelet activation was pronounced, with platelet–monocyte aggregates and platelet-associated TF elevated in DLBCL and FL but not HL. P-selectin was increased in lymphomas with thrombosis, aligned with Khorana and ThroLy scores, and reflected clinical stage while inversely correlating with IPI in non-thrombotic lymphomas. These findings highlight distinct thromboinflammatory mechanisms across lymphoma subtypes, providing insights into biomarkers for thrombosis risk and therapeutic targets in lymphoma management.

## 1. Introduction

Venous thromboembolism (VTE), comprising deep vein thrombosis and pulmonary embolism, is a well-established adverse event in cancer patients, including those with lymphoma. Non-Hodgkin lymphoma is the most frequent hematological malignancy globally, accounting for nearly 3% of cancer diagnoses and deaths [1]. The risk of VTE is elevated during the first two months after lymphoma diagnosis and decreases over time [2]. Diffuse large B-cell lymphoma (DLBCL) and follicular lymphoma (FL) are the most common aggressive and indolent lymphoma subtypes, respectively [3]. The incidence of these lymphomas is expected to rise further [4]. DLBCL, the most common non-Hodgkin lymphoma subtype, is associated with a higher risk of VTE compared to FL [5]. VTE is diagnosed in 11% of patients with DLBCL [6]. The International Prognostic Index (IPI) has been found to be significantly associated with VTE risk in DLBCL, unlike the Khorana score [7]. A meta-analysis has indicated that the overall incidence rate for thrombosis in lymphoma patients is 6.4%, with non-Hodgkin lymphoma patients exhibiting a higher incidence compared to patients with Hodgkin lymphoma (HL) (6.5% vs. 4.7%) [8]. In an additional report, the incidence of thrombotic events is 3.3% in HL, with 90.7% of these being VTE [9].

Recent study has shown that the thrombotic and inflammatory biomarker profiles in lymphoma patients differ significantly from those in healthy volunteers [10]. Notably, endothelial procoagulant tissue factor (TF) and neutrophil extracellular traps (NETs), both of which promote inflammation and thrombosis, have been detected in HL [11]. Additionally, increased inflammatory cytokines, such as interleukin (IL)-6 and IL-10, have been associated with the development of VTE in patients with DLBCL [7]. Furthermore, the anti-inflammatory cytokine IL-10 has been implicated in non-Hodgkin lymphoma risk [12]. Genetic factors may also contribute to lymphoma susceptibility and thrombotic risk. A haplotype comprising single nucleotide polymorphisms in the pro-inflammatory cytokine tumor necrosis factor-alpha (TNF-α) has been linked to an increased risk of non-Hodgkin lymphoma, particularly for DLBCL [13]. Moreover, the IL-10-3575T > A polymorphism has been associated with a heightened risk of both DLBCL and FL [14].

Lymphoma develops in the background of persistent chronic inflammation [15]. To connect chronic inflammation with thrombosis in lymphomas, we explore the profile of inflammatory cytokines and coagulation factors in the peripheral blood of patients with lymphoma, but preferentially patients with DLBCL and FL as non-Hodgkin lymphoma as well as HL. Their expression is correlated with laboratory and clinical parameters of patients with lymphoma. Moreover, the inflammatory cytokines and coagulation factors are compared between the patients with or without thrombosis and healthy volunteers. Chronic inflammation is also examined through the biomarkers of NETs. The predisposition for thrombotic events is evaluated by platelet–monocyte aggregates and circulating coagulation microparticles as well as platelets’ activation in lymphomas. The resulting combination of inflammatory cytokines and coagulation factors may determine the predisposition of lymphomas to thrombotic events.

## 2. Results

### 2.1. Expression of Inflammatory and Coagulation Factors in Lymphomas

**Figure 1:**
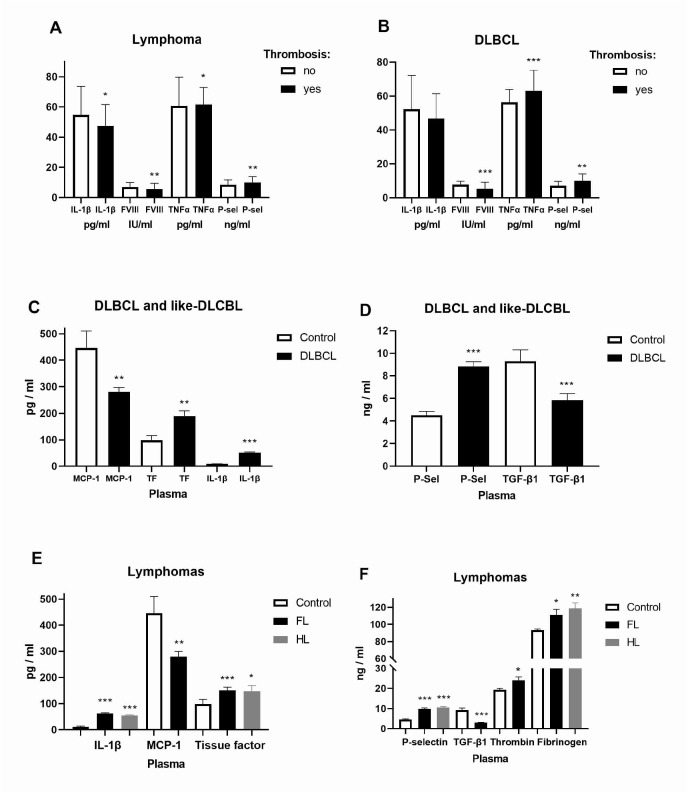
Expression of inflammatory and coagulation factors in lymphomas. Levels of inflammatory factors interleukin (IL-1β), tumor necrosis factor (TNF)-α, tumor growth factor (TGF)-β, monocyte chemoattractant protein-1 (MCP-1) as well as coagulation factors FVIII, tissue factor (TF), P-selectin, thrombin, and fibrinogen in plasma of (**A**) lymphomas without (*n* = 106) or with thrombosis (*n* = 33); (**B**) diffuse large B-cell lymphoma (DLBCL) without (*n* = 43) or with thrombosis (*n* = 24); (**C**,**D**) DLBCL and DLBCL-like lymphomas: T-cell/histiocyte-rich large B-cell lymphoma (n = 1); Burkitt’s Lymphoma (*n* = 3); primary mediastinal large B-cell lymphoma (*n* = 5); mediastinal gray zone lymphoma (*n* = 1); (**E**,**F**) follicular lymphoma (*n* = 28) and Hodgkin lymphoma (*n* = 30) and healthy volunteers (Control, *n* = 18). Values are mean ± SEM. * *p* < 0.05, ** *p* < 0.01, *** *p* < 0.001 vs. appropriate Control or patients with thrombosis (**A**,**B**).

To observe the risk of thrombosis in hematologic malignancies, we examined the levels of inflammatory factors IL-1β, TNF-α, TGF-β, and MCP-1 as well as coagulation factors FVIII, TF, P-selectin, thrombin, and fibrinogen in the plasma of patients with lymphoma. In contrast to IL-1β and FVIII, TNF-α and P-selectin were increased in lymphomas and DLBCL with thrombosis (Figure 1A,B). Contrary to MCP-1 and anti-inflammatory TGF-β, we have increased IL-1β (5.2-fold), TF, and P-selectin levels (2-fold) in joined DLBCL and DLBCL-like lymphomas with an aggressive form of development (Figure 1C,D). MCP-1 and TGF-β (3.1-fold) were also reduced in FL, while thrombin was increased (Figure 1E,F). IL-1β (up to 5.2-fold), P-selectin (2.4-fold), TF, and fibrinogen were generally increased in FL and HL compared to healthy volunteers (Figure 1E,F). As a platelet activation marker, P-selectin was generally increased in lymphomas with thrombosis, while pro-inflammatory cytokine IL-1β was largely increased in DLBCL, FL, and HL. We performed a correlation of the observed inflammatory cytokines with coagulation/clotting factors as well as with blood and clinical parameters for thrombotic events (Table 1). In contrast to pro-inflammatory cytokine IL-1β (r = −0.213), a marker of platelet activation P-selectin was in positive correlation (r = 0.169) with the Khorana score that predicts a risk of VTE for cancer patients as well as with the Thrombosis Lymphoma (ThroLy, r = 0.184) predictive score for assessing the probability of thromboembolic events in patients with lymphoma (Table 1). The ThroLy score was negatively correlated with thrombosis time regarding therapy (ρ = −0.5323, 95% CI = −0.7754—−0.1518, *p* = 0.0074) revealing a higher ThroLy score linked with thrombosis before the start of therapy. Thrombin was in positive correlation (ρ = 0.244) with prothrombin time (PT%). Regarding DLBCL patients’ daily living abilities, the ECOG Scale of Performance Status (ECOG PS) was in negative correlation with IL-8 in patients without thrombosis and with Fibrinogen (ρ = −0.468), TNF-α (ρ = −0.624) and P-selectin (ρ = −0.424) in patients with thrombosis (Table 1). Bulky disease was in positive correlation with FVIII (ρ = 0.335, Table 1) and TF (ρ = 0.369, 95% CI = 0.069–0.61, *p* = 0.0148) in DLBCL without thrombosis. Bulky disease was in positive correlation with P-selectin (ρ = 0.1963, 95% CI = 0.06–0.378, *p* = 0.044) in lymphomas without thrombosis. ECOG PS was in positive correlation with FVIII (ρ = 0.2237, 95% CI = 0.029–0.402, *p* = 0.021) and thrombin (ρ = 0.199, 95% CI = 0.003–0.38, *p* = 0.041) in lymphomas without thrombosis. P-selectin was in positive correlation (ρ = 0.205) with the clinical stage of lymphomas without thrombosis, but in negative correlation (ρ = −0.357) with the International Prognostic Index (IPI) as a scoring system for aggressive non-Hodgkin lymphoma (Table 2). TF was in positive correlation (ρ = 0.228) with the clinical stage of lymphomas without thrombosis (Table 2). TNF-α was generally in negative correlation with IPI in lymphomas (ρ = −0.448) and DLBCL (ρ = −0.426) with thrombosis (Table 2). Lactate dehydrogenase (LDH) was in positive correlation with the clinical stage (ρ = 0.283, 95% CI = 0.079–0.464, *p* = 0.006), IPI (ρ = 0.356, 95% CI = 0.159–0.525, *p* = 0.0004), bulky disorder (ρ = 0.3158, 95% CI = 0.079–0.464, *p* = 0.002), and ECOG PS (ρ = 0.2076, 95% CI = −0.001–0.399, *p* = 0.045), but in negative correlation with IL-8 (ρ = −0.3142, 95% CI = −0.568–−0.006, *p* = 0.04) in DLBCL without thrombosis. Bulky disorder was in positive correlation with TF (ρ = −0.369, 95% CI = 0.069–0.609, *p* = 0.015). Therefore, P-selectin and TF were predictors of clinical stage progression, while TNF-α was predictor of IPI in lymphomas.

### 2.2. Inflammatory Response Through Neutrophil Extracellular Traps in Lymphomas

**Figure 2:**
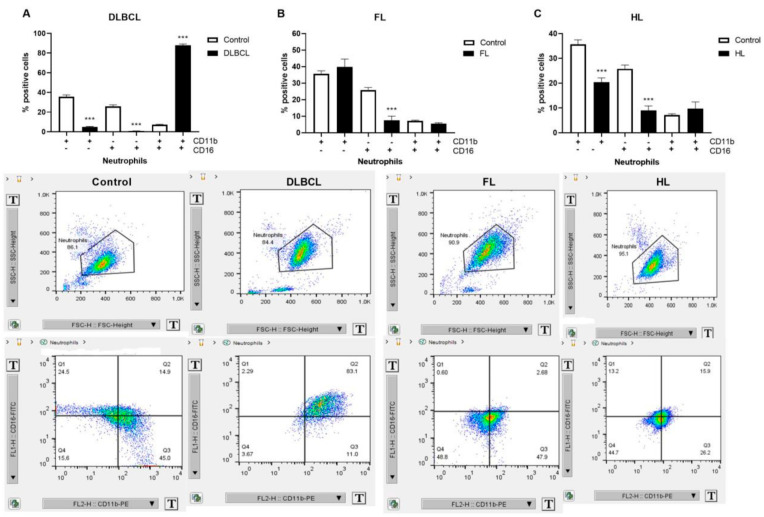
Levels of neutrophils in lymphomas. Using flow cytometry, we determined levels of CD11b and CD18 markers of neutrophiles in (**A**) diffuse large B-cell lymphoma (DLBCL, *n* = 6), (**B**) follicular lymphoma (FL, *n* = 5), and (**C**) Hodgkin lymphoma (HL, *n* = 5) in comparison to healthy volunteers (Control, *n* = 5). Next to the graphs are gating images for CD11b and CD18 markers’ distribution by flow cytometry. Values are mean ± SEM. *** *p* < 0.001 vs. corresponding Control.

**Figure 3:**
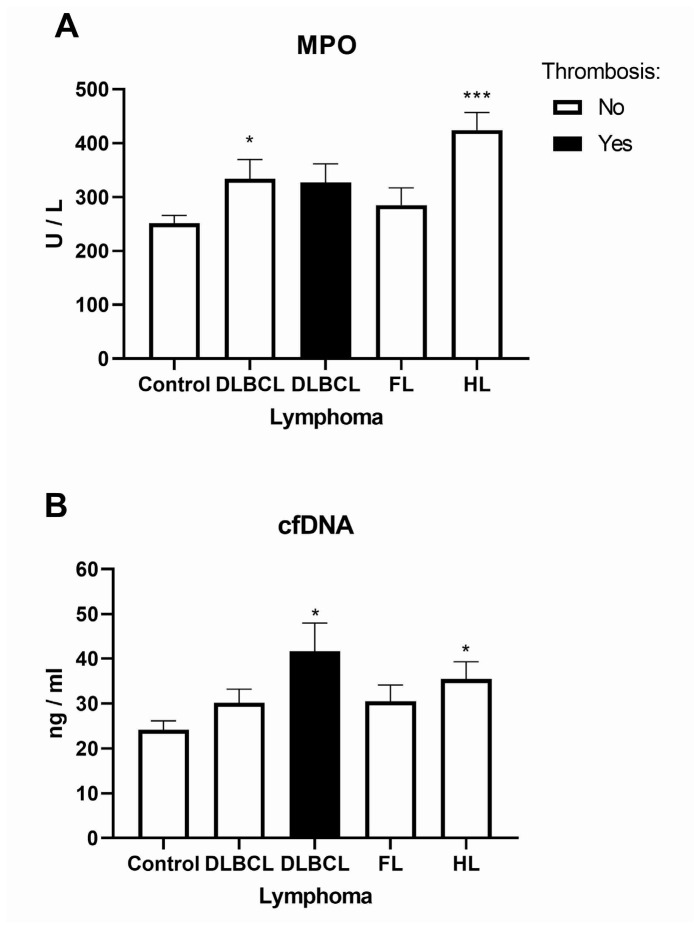
Expression of biomarkers of neutrophil extracellular traps in lymphomas. Level of biomarkers of neutrophil extracellular traps (NETs): (**A**) myeloperoxidase (MPO) and (**B**) cell-free DNA (cfDNA) in plasma of peripheral blood of healthy volunteers (Control, *n* = 27/8, for (**A**)/(**B**)) and patients of follicular lymphoma (FL, *n* = 25/23, for (**A**)/(**B**)), Hodgkin lymphoma (HL, *n* = 32/21, for (**A**)/(**B**)), and diffuse large B-cell lymphoma (DLBCL) with or without thrombosis (*n* = 27/24, for (**A**)/(**B**)). Values are mean ± SEM. * *p* < 0.05, *** *p* < 0.001 vs. Control.

Neutrophils are continuously recruited in the chronic inflammation and contribute through the formation of NETs, as well as the activation of other immune cells [16]. Neutrophil activation markers CD11b/CD16, linked to inflammation, were mutually increased 12-fold in DLBCL patients, but not individually, compared to healthy volunteers (Figure 2A). Levels of individual CD11b and CD16 were generally downregulated in the examined lymphomas: DLBCL, FL, and HL (Figure 2A–C). As biomarkers of NETs, myeloperoxidase (MPO) and cell-free DNA (cfDNA) expression have been examined in lymphomas with or without thrombosis (Figure 3). MPO was elevated in DLBCL patients without thrombosis, while cfDNA was elevated in DLBCL patients with thrombosis (Figure 3A,B). MPO and cfDNA were increased in patients with HL (Figure 3). The cfDNA was in high negative correlation with the ThroLy score (r = −0.588, Table 1). The examined NET biomarkers show divergence with respect to thrombotic events in patients with DLBCL.

### 2.3. The Platelet and Monocyte as Carriers of Circulating Tissue Factor Microparticles in Lymphomas

**Figure 4:**
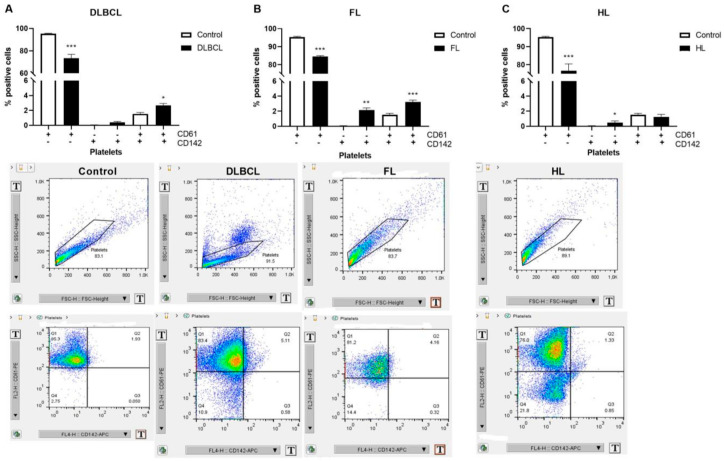
Levels of platelets in lymphomas. Using flow cytometry, we determined the levels of markers, CD61 of platelets and CD142 of tissue factor in (**A**) diffuse large B-cell lymphoma (DLBCL, *n* = 5), (**B**) follicular lymphoma (FL, *n* = 5), and (**C**) Hodgkin lymphoma (HL, *n* = 5) in comparison to healthy volunteers (Control, *n* = 5). Next to the graphs are gating images for CD61 and CD142 markers’ distribution by flow cytometry. Values are mean ± SEM. * *p* < 0.05, ** *p* < 0.01, *** *p* < 0.001 vs. corresponding Control.

**Figure 5:**
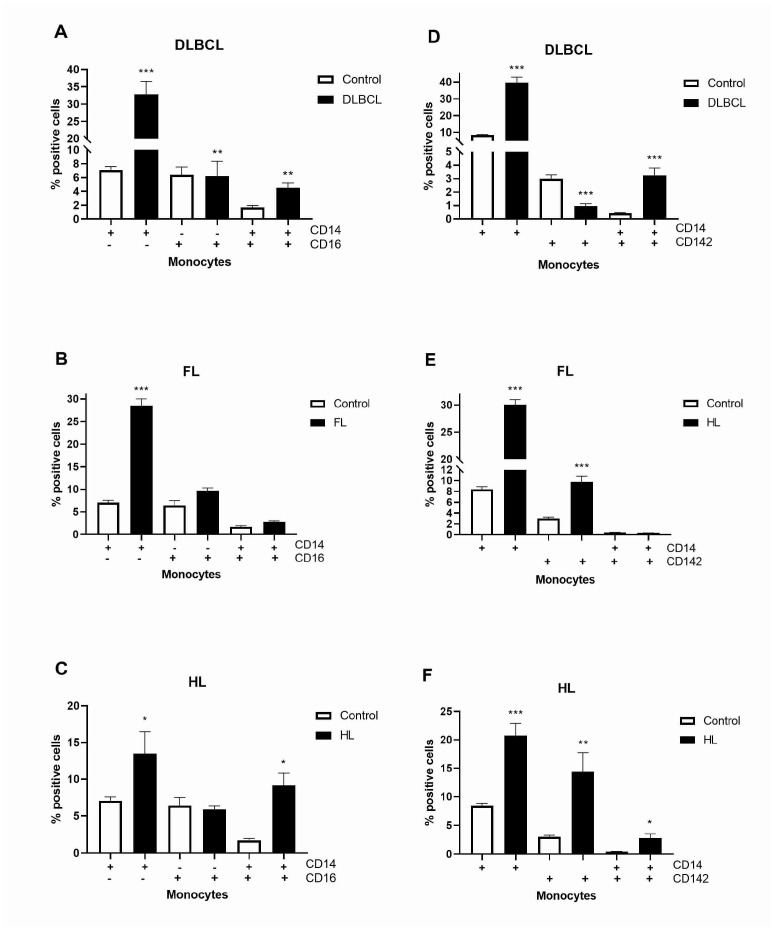
Levels of monocytes in lymphomas. Using flow cytometry, we determined the levels of CD14 and CD16 markers of monocytes in (**A**) diffuse large B-cell lymphoma (DLBCL, *n* = 5), (**B**) follicular lymphoma (FL, *n* = 5), and (**C**) Hodgkin lymphoma (HL, *n* = 5) in comparison to healthy volunteers (Control, *n* = 5). Also, we determined levels of CD14 marker of monocytes and the CD142 marker of tissue factor in (**D**) DLBCL (*n* = 5), (**E**) FL (*n* = 5), and (**F**) HL (*n* = 5) in comparison to the Control (*n* = 5). Values are mean ± SEM. * *p* < 0.05, ** *p* < 0.01, *** *p* < 0.001 vs. corresponding Control.

The platelet–monocyte affiliation has appeared as a main mechanism linking thrombosis and inflammation [17]. As the main cause of fibrin formation, TF (CD142) is one of the most procoagulant circulating microparticles [18]. Patients with lymphomas generally had decreased level of platelets and increased TF compared to healthy volunteers (Figure 4). Patients with non-Hodgkin lymphoma (DLBCL and FL) had increased levels of platelet (CD61+)-derived circulating microparticles carrying TF (CD142+), but not patients with HL (Figure 4). Chronic inflammation was characterized by a continuous recruitment of monocytes that influence multiple aspects of tumor progression [19]. We first explored three major populations of monocytes: classical (CD14+CD16−), non-classical (CD14−CD16+), and intermediate (CD14+CD16+). Classical monocytes were more than 4-fold elevated in patients with DLBCL and FL and 2-fold in HL, while intermediate monocytes linked to inflammation were 2.7-fold elevated in patients with DLBCL and 5.4-fold in HL (Figure 5A–C, Supplemental Figure S1). Patients with DLBCL and HL had about 7-fold increased levels of monocyte (CD14+)-derived circulating microparticles carrying TF (CD142+, Figure 5D,F). The levels of TF were reduced in DLBCL but increased 3.3-fold in FL and 4.8-fold in HL (Figure 5D–F). We demonstrated the increased levels of platelet- and monocyte-carrying procoagulant TF related to inflammation in lymphomas.

### 2.4. Platelet–Monocyte Aggregates and Platelets Activation in Lymphomas

**Figure 6:**
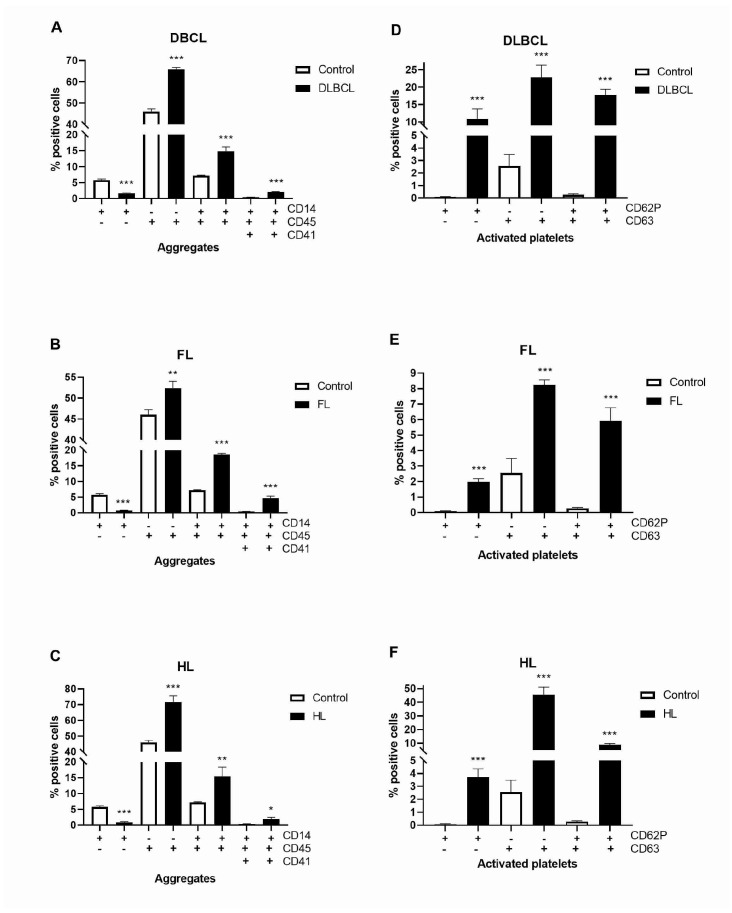
Level of platelet–monocyte aggregates and activated platelets in lymphomas. Using flow cytometry, we determined markers of platelet–monocyte aggregates (CD14+, CD45+, CD41+) and platelet activation (CD62P+/CD63+) in (**A**) diffuse large B-cell lymphoma (DLBCL, *n* = 4), (**B**) follicular lymphoma (FL, *n* = 5), and (**C**) Hodgkin lymphoma (HL, *n* = 5) in comparison to healthy volunteers (Control, n = 5). Also, we determined markers of platelets activation (CD62P+/CD63+) in (**D**) DLBCL (*n* = 5), (**E**) FL (*n* = 4), and (**F**) HL (*n* = 5) compared to the Control (*n* = 4). Values are mean ± SEM. * *p* < 0.05, ** *p* < 0.01, *** *p* < 0.001 vs. corresponding Control.

Monocytes were recognized as CD14+ and the CD45+CD14+ were further analyzed for CD41+ and CD41- events, where CD41 was expressed by platelets and CD45 was expressed on all leukocytes. CD14+CD45+ monocytes were more than double increased, while platelet–monocyte aggregates (CD14+CD45+CD41+) were 5–10-fold increased in lymphomas than in healthy volunteers (Figure 6A–C, Supplemental Figure S2). CD63 and CD62P (P-selectin) have been accepted as platelet activation markers. Mutually and individually, they have been largely increased in lymphomas (Figure 6D–F, Supplemental Figure S3). Activated CD63+CD62P+ platelets were 66-, 22- and 33-fold increased in DLBCL, FL, and HL, respectively, compared to healthy volunteers. The combination of increased platelets activation and platelet–monocyte aggregates generally supports the predisposition of lymphomas to thrombosis.

## 3. Discussion

This study provides novel insights into the thromboinflammatory mechanisms underlying lymphomas, with a focus on pro-inflammatory cytokines, neutrophil, and monocyte activation, and platelet function as biomarkers for thrombosis risk. These findings emphasize the complex interplay between inflammation and coagulation factors in lymphoma pathogenesis. The incidence of VTE was higher in patients with NHL (8.0%) than HL (6.7%), and it is in accordance with the presented predominance of thrombosis in patients with DLBCL [20].

The observed increase in IL-1β across lymphomas underscores its role in promoting a pro-inflammatory microenvironment that supports coagulation activation. Its inverse correlation with the Khorana score for VTE prediction suggests that inflammation-driven thrombosis in lymphomas may bypass traditional risk assessment models. IL-1β is known to enhance endothelial activation, increase TF expression, and amplify coagulation cascades [21]. The AG genotype of IL-1β rs3917356 decreased the risk of NHL and increased risk of HL [22]. In response to tissue injury, IL-1β and coagulation FVIII are increased, but we demonstrated their lower levels that question the relevance of tissue injury in thrombosis of lymphomas. Previous study showed high levels of IL-10, IL-6, IL-1β, and TNF-α in lymphoma patients except TNF-α in NHL patients [23]. TNF-α acts as a main inducer of endothelial activation, neutrophil accumulation and activation, and platelet adhesion and aggregation, linking inflammation and the hypercoagulable state [24]. We showed that pro-inflammatory TNF-α was elevated in lymphomas with thrombosis and inversely associated with the IPI. Patients with lower IPI scores already have largely increased TNF-α in lymphomas with increased risk for thrombosis.

Neutrophil activation was increased in presented DLBCL patients. NETs have been implicated in thrombus formation through their capacity to trap platelets, promote TF activity, and amplify coagulation [25]. The inverse association of MPO with thrombotic events and the alignment of cfDNA with thrombosis but not the ThroLy score suggests that NET biomarkers may act as independent drivers of thrombosis, consistent with previous reports showing elevated NET markers in cancer-associated thrombosis and their potential as predictive biomarkers [26,27]. Moreover, eight NET-related genes were found to have predictive potential for DLBCL patient survival [28]. Plasma and tumor tissue levels of NETs and MPO–DNA were higher in advanced-stage DLBCL and correlate with inferior survival [29]. We demonstrated that MPO and cfDNA were increased in patients with HL. Particularly, only a nodular sclerosis subtype of HL exhibited NET formation [11].

Monocyte subsets further elucidate the inflammatory milieu in lymphomas. We presented that classical monocytes were elevated across all examined lymphoma subtypes, while intermediate monocytes, known for their inflammatory and procoagulant properties, were increased in DLBCL and HL with particularly elevated TF-carrying intermediate monocytes. The levels of TF were reduced in DLBCL but increased in FL and HL. TF expression was reduced in presented patients with DLBCL while elevated in FL and HL, correlating with lymphoma stage. These observations are consistent with studies demonstrating monocyte-derived TF as a key mediator of cancer-associated hypercoagulability [30].

TF and fibrinogen were increased in FL and HL, while TF levels were in correlation with the clinical stage of lymphomas without thrombosis. According to a previous report, the plasma fibrinogen level did not have prognostic significance for DLBCL patients, but it was correlated with clinical features and laboratory parameters [31]. However, another report revealed contradictory information, showing that increased fibrinogen levels at diagnosis predict poor outcomes in patients with DLBCL in accordance with our results [32]. Thrombin was increased in FL, and in accordance with prothrombin time in DLBCL without thrombosis. The peak thrombin levels were decreased in the lymphoma patients and similar in HL and NHL [33].

Platelet dysfunction emerged as a hallmark of lymphoma-associated thrombosis, with significant increases in platelet–monocyte aggregates and platelet-associated TF in DLBCL and FL. Platelet activation markers and P-selectin were consistently elevated in examined lymphomas, the latest particularly in cases with thrombosis. P-selectin, a marker of platelet degranulation, facilitates platelet–leukocyte interactions and contributes to thrombus stability [34]. A previous study revealed that P-selectin was elevated in patients with NHL and HL [35]. Its alignment with the clinical stage and predictive scores such as the Khorana and ThroLy underscores its utility as a biomarker for thrombosis risk stratification in lymphoma patients. The reduction in platelet counts observed across lymphomas may reflect increased platelet consumption and sequestration in thromboinflammatory processes. Moreover, the prothrombin time in DLBCL without thrombosis and elevated thrombin levels in FL suggest subtype-specific coagulation dynamics, supporting distinct thromboinflammatory profiles in lymphomas.

In conclusion, this study highlights distinct thromboinflammatory profiles across lymphoma subtypes. IL-1β, TNF-α, NET biomarkers, and P-selectin emerge as key players, with potential applications in risk prediction and targeted therapy. These findings may inform personalized approaches for thrombosis risk stratification and management in lymphoma patients. Future research should explore the mechanistic underpinnings of these associations and evaluate therapeutic strategies aimed at disrupting thromboinflammatory pathways. Anti-inflammatory agents targeting IL-1β or TNF-α, along with anticoagulants, may represent promising avenues for reducing thrombosis risk in lymphoma patients.

## 4. Materials and Methods

### 4.1. Patients’ Characteristics

In the study, 156 newly diagnosed patients with lymphoma were prospectively recruited from the Clinic of Hematology, University Clinical Center of Serbia, Belgrade, Serbia. Clinical follow-up included laboratory data: full blood count, D-dimer, prothrombin time (PT—extrinsic/common pathways), international normalized ratio (INR), activated partial thromboplastin time (aPTT—intrinsic/common pathways), thrombin time (TT), fibrinogen, C-reactive protein (CRP), ferritin, erythrocyte sedimentation rate (ESR), and previous thromboembolic events. Informed consent was obtained from all of the participants included in the study, approved by the clinical and institutional ethical committees.

### 4.2. Isolation and Preparation Cells from Peripheral Blood

Plasma, mononuclear cells (MNCs), granulocytes, and platelets were isolated from peripheral blood (30 mL) of patients with DLBCL, HL, and FL. Blood was collected in tubes with ethylenediaminetetraacetic acid (EDTA, BD Vacutainer K2A) and separated by Lymphocyte Cell Separation Media (Capricorn Scientific, Ebsdorfergrund, Germany) gradient centrifugation. MNCs and granulocytes were used to quantify the specific CD markers on the surface of monocytes and neutrophils, respectively, by flow cytometry.

### 4.3. ELISA Assay

Quantification of the inflammatory interleukin (IL)-1β, tumor necrosis factor (TNF)-α, monocyte chemoattractant protein-1 (MCP-1), IL-8, tumor growth factor (TGF)-β1, and coagulation parameters like soluble P-selectin, FVIII, thrombin, fibrinogen and circulating TF from the patient’s and healthy donor’s plasma samples was performed using ELISA kits (Elabscience Biotechnology Inc., Houston, TX, USA) according to the manufacturer’s instructions. All plasma samples were tested in triplicate and the results were obtained from a standard curve that was created using recombinant standards and expressed as the average concentration of each tested cytokine and coagulation parameter in adequate units. The measurements were performed on an ELISA Multiscan Plus plate reader (Labsystems, Vantaa, Finland).

### 4.4. Determination of NET Quantity from the Plasma of the Patients and Healthy Controls

MPO activity and cfDNA concentration were examined in plasma of peripheral blood samples from patients of follicular lymphoma (25/23), Hodgkin lymphoma (32/21), diffuse large B-cell lymphoma (DLBCL) with or without thrombosis (27/24), and healthy volunteers (27/8). For the neutrophil extracellular traps study (NETs), blood samples were collected in tubes treated with EDTA. The plasma was separated by centrifugation and used to analyze the NETs markers: the concentration of circulating DNA (Circulating DNA Quantification Kit, Abcam, Cambridge, UK) and myeloperoxidase activity (MPO, Myeloperoxidase Activity Assay Kit, Elabsciences Biotechnology Inc.). Fluorescence was measured using a Perkin Elmer Wallac 1420 Victor2 instrument (Perkin Elmer, Buckinghamshire, UK), and absorbance was read using an ELISA microplate reader (RT-6100, Raito, Shenzhen, China).

### 4.5. Flow Cytometry

The expression of the CD markers on the peripheral blood cell surface was analyzed by flow cytometry. MNC, granulocytes, and platelets were fixed in 4% paraformaldehyde (Thermo Scientific, Rockford, IL, USA) and stained with fluorescein isothiocyanate (FITC)-, phycoerythrin (PE)-, allophycocyanin (APC)-, or peridinin chlorophyll protein complex/cyanine5.5 (PerCP/Cy5.5)-conjugated antibodies directed against human CD14-PE (Elabscience, FW1347), CD16-FITC (Elabscience, 225367, Houston, TX, USA), and CD142-APC (BioLegend 365206, San Diego, CA, USA) for monocytes; CD61-PE (Elabscience, 225359, Houston, TX, USA) for platelets, CD11b-PE (Elabscience, 225358, Houston, TX, USA) for neutrophils; CD45-FITC (BioLegend, 304054, San Diego, CA, USA) and CD41-PerCP/Cy5.5 (BioLegend, 303720) for monocyte/platelet aggregates; and CD62P-FITC (BioLegend, 304904, San Diego, CA, USA) and CD63-PE (BioLegend, 353004, San Diego, CA, USA) for activated platelets. Labeled cells were analyzed using BD FACS Calibur (BD Bioscience, Franklin Lakes, NJ, USA). For each sample, at least 10,000 events were recorded.

### 4.6. Statistical Analysis

The normality of data distribution was examined by the Shapiro–Wilk and Kolmogorov–Smirnov tests. The results are expressed as mean ± SEM. Differences between groups were analyzed using Student’s *t*-test of the Prism 6 software (GraphPad Software Inc., San Diego, CA, USA). When the distribution was not normal, the Mann–Whitney test was used for intergroup comparisons. The correlations between numerical variables were assessed by Spearman’s or Pearson’s correlation coefficients depending on the normality of data distribution. *p* < 0.05 was considered statistically significant.

## Figures and Tables

**Table 1:** Correlation of inflammatory and coagulation cytokines with clinical parameters of patients with DLBCL (no thrombosis n = 43 and with thrombosis n = 24).

Spearman or Pearson		HGB/ PT-s	MCV/ aPTT	MCH/ β2M	MCHC/MPV	RDW/ PDW	PLT/ ECOG PS	PCT/ Bulky	INR/ Khorana Score	PT%/ ECOG PS	Fibrinogen	ESR/ThroLy Score	NLR/ CRP	PLR
**IL-1β**	ρ/r	0.203	0.17	0.259	0.324	−0.256	−0.217	−0.231	**−0.213**		−0.214	−0.246	**−0.175**	−0.22
	95% CI	0.032–0.36	−0.002–0.33	0.09–0.41	0.16–0.47	−0.41–−0.09	−0.37–−0.05	−0.39–−0.06	**−0.37–−0.04**		−0.37–−0.04	−0.4–−0.076	**−0.34–−0.003**	−0.38–−0.05
	*p* value	0.017	0.046	0.002	0.0001	0.002	0.011	0.006	**0.013**		0.012	0.004	**0.04**	0.01
**FVIII**	ρ/r	0.175	0.214	0.267	0.254	−0.278		**0.335**						
	95% CI	0.003–0.337	0.043–0.37	0.1–0.42	0.086–0.41	−0.43–−0.11		**0.029–0.58**						
	*p* value	0.04	0.012	0.002	0.003	0.001		**0.028**						
**IL-8**	ρ/r		**−0.214**	**0.245**	**0.237**	**0.204**	−0.301	−0.213		**−0.302**	−0.216		−0.181	−0.235
	95% CI		**−0.37–−0.04**	**0.07–0.4**	**0.07–0.39**	**0.03–0.36**	−0.45–−0.14	−0.37–−0.04		**−0.559–0.007**	−0.37–−0.045		−0.34–−0.009	−0.39–−0.07
	*p* value		**0.012**	**0.004**	**0.005**	**0.017**	0.0003	0.012		**0.049**	0.0111		0.0336	0.0055
**Fibrinogen**	ρ/r		**−0.24**		**−0.236**	0.1977				−0.468 *				
	95% CI		**−0.4–−0.07**		−0.39–−0.066	0.03–0.36				**−0.744–−0.057**				
	*p* value		**0.0045**		0.0054	0.0201				**0.0242**				
**TNF-α**	ρ/r			−0.185	−0.173	**−0.204**	0.2617	0.2699		−0.624 *				
	95% CI			−0.35–−0.013	−0.34–−0.001	**−0.36–−0.032**	0.09–0.42	0.1–0.42		**−0.828–−0.274**				
	*p* value			0.0301	0.0422	**0.017**	0.0019	0.0014		**0.0014**				
**P-selectin**	ρ/r	**0.168**	−0.192	−0.275	−0.288	0.272	**−0.424**		0.169	−0.211		**0.184**		
	95% CI	**−0.01–0.33**	−0.35–−0.021	−0.43–−0.11	−0.44–−0.12	0.1–0.42	**−0.718–−0.001**		−0.003–0.33	−0.37–−0.04		**0.012–0.35**		
	*p* value	**0.0496**	0.024	0.0011	0.0006	0.0013	**0.043**		0.047	0.0145		**0.0314**		
**MCP-1**	ρ/r	−0.191			−0.178							0.1775		
	95% CI	−0.35–−0.02			−0.34–−0.006							0.004–0.34		
	*p* value	0.0249			0.0366							0.0394		
**Thrombin**	ρ/r	**−0.24**	0.222	0.2391			−0.245	−0.21	−0.216	0.244	−0.222	−0.214	−0.305	−0.313
	95% CI	**−0.4–−0.07**	0.05–0.38	0.0–0.39			−0.4–−0.08	−0.37–−0.04	−0.37–−0.05	0.07–0.4	−0.380.05	−0.37–−0.04	−0.45–−0.14	−0.46–−0.15
	*p* value	**0.0045**	0.0089	0.0047			0.0038	0.0137	0.01	0.0044	0.0087	0.0128	0.0003	0.0002
**TGF-β1**	ρ/r						0.264	0.261			0.23			
	95% CI						0.05–0.46	0.045–0.45			0.013–0.43			
	*p* value						0.0142	0.0157			0.033			
**MPO**	ρ/r		0.55 *	−0.56 *										
	95% CI		0.05–0.83	**−0.84–−0.05**										
	*p* value		0.035	**0.032**										
**cfDNA**	ρ/r				**−0.46**	**−0.44**	**0.447**	0.355				**−0.588**		0.429
	95% CI				**−0.68–−0.16**	**−0.67–−0.122**	**0.13–0.679**	0.02–0.615				**−0.85–−0.108**		0.11–0.667
	*p* value				**0.004**	**0.007**	**0.006**	0.031				**0.021**		0.008

Bolded values correspond to bolded parameters. * patients with thrombosis. Prokalcitonin (PCT), neutrophils–lymphocyte ratio (NLR), platelet–lymphocyte ratio (PLR), and the C-reactive protein (CRP), international normalized ratio (INR), erythrocyte sedimentation rate (ESR), platelets (PLT), Beta-2 microglobulin (β2M), mean corpuscular hemoglobin (MCH), Mean Corpuscular Hemoglobin Concentration (MCHC), Mean Platelet Volume (MPV), platelet distribution width (PDW), red cell distribution width (RDW), Mean Corpuscular Volume (MCV), activated partial thromboplastin time (aPTT), hemoglobin (HGB), prothrombin time (PT) in seconds and %, ECOG Scale of Performance Status (ECOG PS).

**Table 2:** Correlation of inflammatory and coagulation cytokines with clinical stage and prognosis of patients with lymphoma and DLBCL in accordance with thrombosis.

	Spearman	P-Selectin	Tissue Factor	Fibrinogen	TNF-α	MPO
Lymphoma (no thrombosis n = 106 and with thrombosis n = 33)						
Clinical stage	ρ	0.205	0.228		**−0.372**	
	95% CI	0.01–0.39	0.03–0.41		**−0.65–−0.009**	
	*p* value	0.035	0.018		**0.039**	
International Prognostic Index (IPI)	ρ	**−0.357**		−0.242	**−0.448**	
	95% CI	**−0.64–0.008**		−0.42–−0.05	**−0.698–−0.1**	
	*p* value	**0.049**		0.012	**0.012**	
DLBCL (no thrombosis n = 43 and with thrombosis n = 24)						
Clinical stage	ρ				−0.34	0.466
	95% CI				−0.587–−0.035	0.12–0.71
	*p* value				0.026	0.008
International Prognostic Index (IPI)	ρ				**−0.426**	
	95% CI				**−0.714–−0.014**	
	*p* value				**0.038**	

Bolded values are lymphoma/diffuse large B-cell lymphoma (DLBCL) with thrombosis. Myeloperoxidase (MPO), tumor necrosis factor (TNF)-α.

## Data Availability

The data are not publicly available due to privacy and ethical restrictions.

## Supplementary Materials

The following supporting information can be downloaded at https://www.mdpi.com/article/10.3390/ijms26052058/s1.
