# Supplementary material for: Thromboinflammatory Biomarkers in Lymphomas: Linking Inflammation to Thrombosis Risk
Source: Int J Mol Sci. 2025 Feb 26;26(5):2058. doi: 10.3390/ijms26052058 (PMC11900196; doi:10.3390/ijms26052058)
Supplement: Supplementary file 1 [file ijms-26-02058-s001.zip › ijms-3423892-supplementary.pdf]

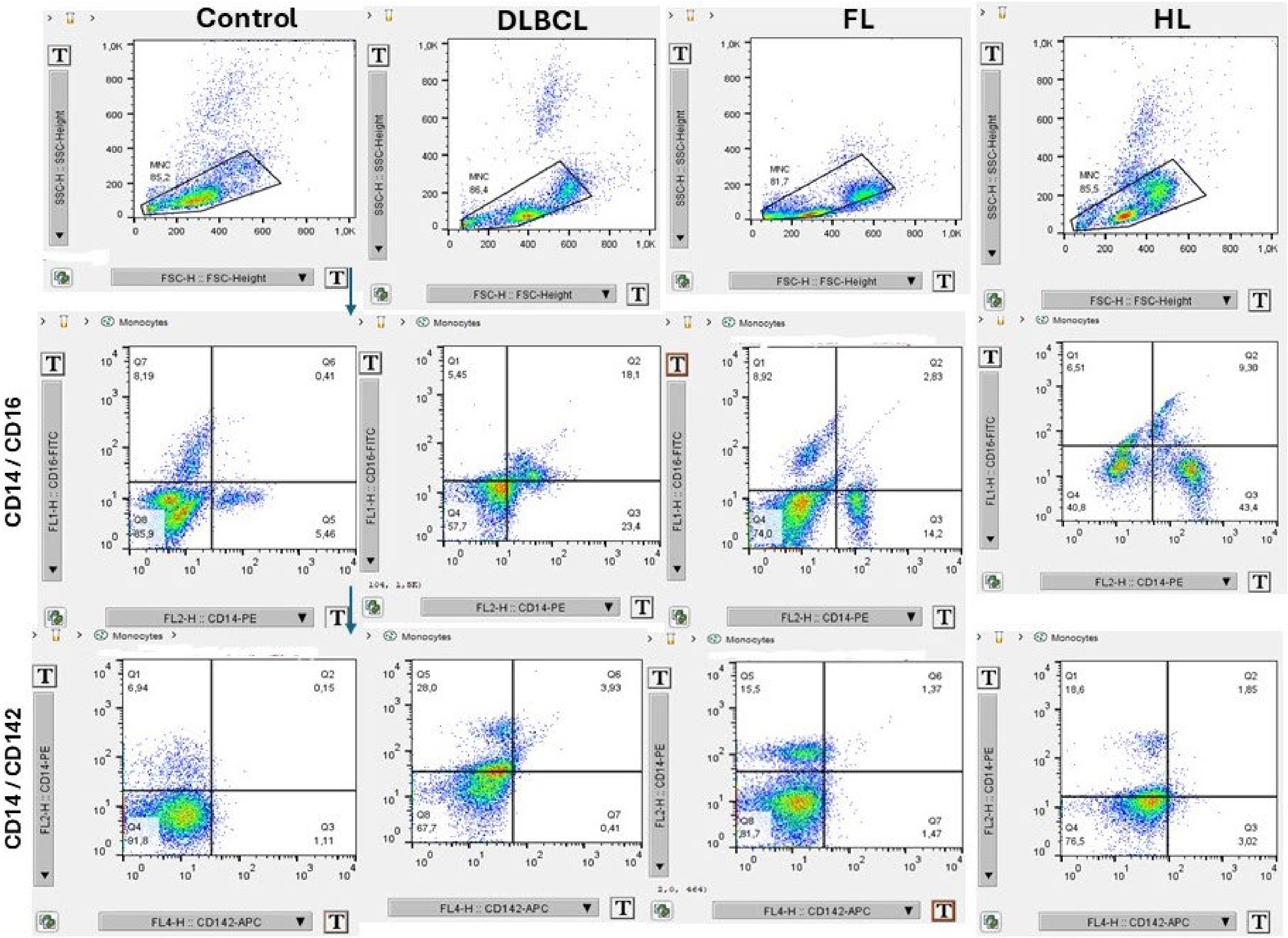

Supplemental figure 1. Levels of monocytes in lymphomas. Using flow cytometry, we determined levels of CD14 and CD16 markers of monocytes in diffuse large B-cell lymphoma (DLBCL), follicular lymphoma (FL) and Hodgkin lymphoma (HL) in comparison to healthy volunteers (Control). These gating images for CD14, CD16 and CD142 markers distribution, determined by flow cytometry, correspond to Figure 5.

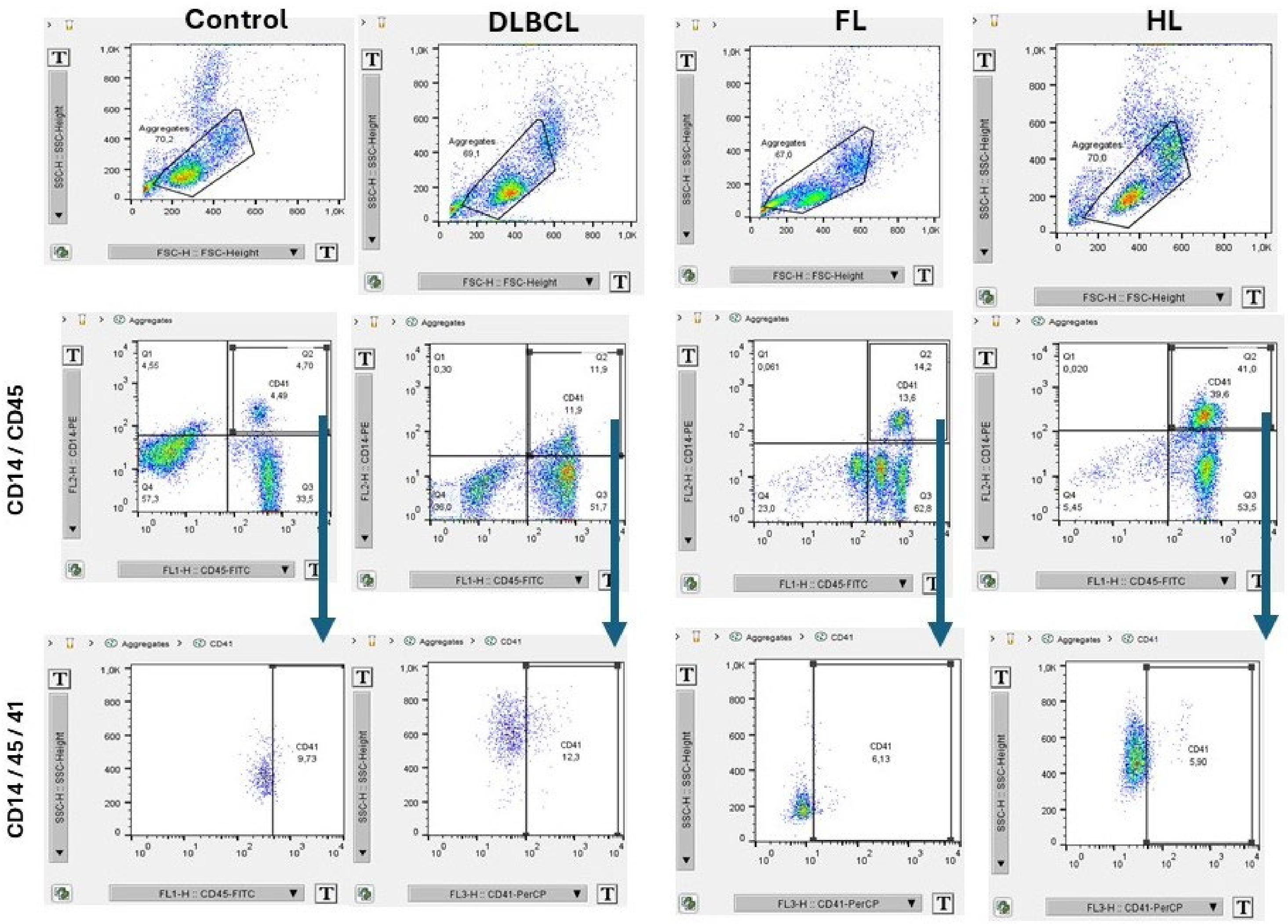

Supplemental figure 2. Levels of platelet-monocyte aggregates in lymphomas. Using flow cytometry, we determined levels of CD14, CD45 and CD41 markers of platelet-monocyte aggregates in diffuse large B-cell lymphoma (DLBCL), follicular lymphoma (FL) and Hodgkin lymphoma (HL) in comparison to healthy volunteers (Control). These gating images for CD14, CD45 and CD41 markers distribution, determined by flow cytometry, correspond to Figure 6.

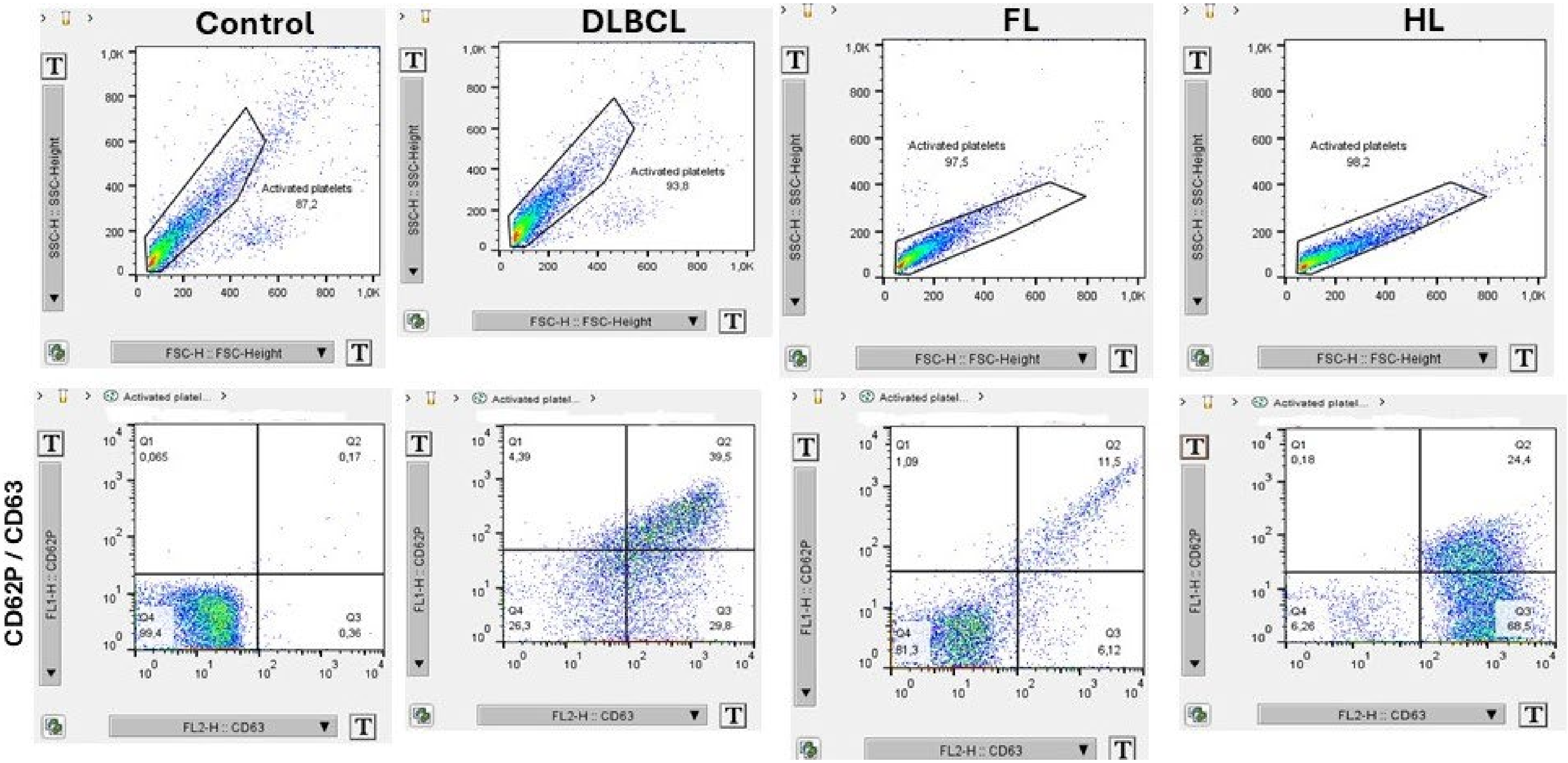

Supplemental figure 3. Levels of platelet activation in lymphomas. Using flow cytometry, we determined levels of CD62P and CD63 markers of platelets activation in diffuse large B-cell lymphoma (DLBCL), follicular lymphoma (FL) and Hodgkin lymphoma (HL) in comparison to healthy volunteers (Control). These gating images for CD62P and CD63 markers distribution, determined by flow cytometry, correspond to Figure 6.
